# Review of the Effects of Antibiotics on Nitrogen Cycle and Greenhouse Gas Emissions in Aquaculture Water

**DOI:** 10.3390/toxics14010043

**Published:** 2025-12-30

**Authors:** Hanxiao Wang, Lan Zhang, Shicheng Zhang, Haoyan Li, Changyan Sun, Yan Wang, Xiaoshuai Hang

**Affiliations:** 1 Nanjing Institute of Environmental Sciences, Ministry of Ecology and Environment, Nanjing 210042, China; 2 Key Laboratory of Integrated Regulation and Resource Development on Shallow Lakes, Ministry of Education, College of Environment, Hohai University, Nanjing 210098, China

**Keywords:** nitrification, denitrification inhibition, ecological risk, antibiotic resistance genes, greenhouse gases, microbial adaptation

## Abstract

Aquaculture systems face escalating ecological risks due to the widespread use and persistence of antibiotics, which disrupt microbial-mediated nitrogen cycling and exacerbate greenhouse gas (GHG) emissions. This review synthesizes the recent research on how common antibiotics, such as sulfonamides, quinolones, tetracyclines, and macrolides, with the concentration ranging from μg/L to mg/L, alter microbial community structure, functional gene expression (e.g., *amoA*, *nirK*, and *nosZ*), and key nitrogen transformation processes. These disruptions inhibit nitrogen-removal efficiency by 25–55%, promote the accumulation of toxic intermediates (e.g., NH_4_^+^ and NO_2_^−^), and enhance emissions of potent GHGs of nitrous oxide (N_2_O) and methane (CH_4_). The effects are influenced by antibiotic type; concentration; environmental conditions; and interactions with co-contaminants such as heavy metals (Cu^2+^ and Pb^2+^ at 50–200 μg/L) and microplastics (0.1–10 mg/L), which can synergistically amplify ecological risks by 20–40%. The research in this field has largely focused on the toxicity of individual antibiotics, so significant gaps remain regarding combined pollution effects, long-term microbial adaptation, and molecular-scale mechanisms. This review synthesizes research on the impacts of aquaculture antibiotics on microbial nitrogen cycling and GHG emissions, identifying key mechanisms and research gaps. Its significance lies in laying a scientific foundation for integrated antibiotics pollution control strategies and bridging basic research with practical aquaculture management to advance the sustainability of aquaculture ecosystems.

## 1. Introduction

Antibiotics, defined as chemical agents that either inhibit or induce the death of microorganisms, have been used in human, veterinary, and agricultural medicine since their invention [1]. More than 100,000 tons of antibiotics are globally consumed each year, and it is estimated that 30–90% of these antibiotics and their metabolites are released into the environment, either as parent compounds or metabolites [2]. With the rapid development of aquaculture, antibiotics are extensively used in aquaculture for the prevention and treatment of fish diseases [3]. However, the overuse of these antibiotics can lead to persistent residues in aquaculture water and sediments and can facilitate further development of antibiotic-resistant bacteria due to antibiotic persistence in the environment [4]. Studies showed that in some regions in China, antibiotic concentrations in aquaculture effluents have reached levels of μg/L or, in some cases, mg/L [5]. Chen et al. (2018) assessed antibiotic residues in fish from typical aquaculture regions, examining the distribution, bioaccumulation, and relative health risks [6]. Antibiotics can remain in the ecosystem, evade natural degradation, and gradually bioaccumulate, which threatens ecosystems and human health [7].

Globally, the standards and regulations for antibiotic concentration limits exhibit a multi-level, differentiated, and increasingly stringent pattern across various media and regions [8]. For aquaculture product residues, the EU has set the strictest requirements via Regulation (EU) No. 37/2010 and Decision 2002/657/EC, imposing undetectable thresholds on banned substances like chloramphenicol (0.3 μg/kg) and nitrofurans (1 μg/kg), with defined maximum residue limits (MRLs) for permitted antibiotics (e.g., 200 μg/kg for tetracyclines in muscle) (official website of the European Union, https://aquaculture.ec.europa.eu/, accessed on 21 December 2025). China banned 22 types of antibiotics and specifying MRLs for 104 permitted ones (e.g., 100 μg/kg for enrofloxacin + ciprofloxacin in fish), while the US FDA and Japan’ s Positive List System also establish strict residue controls, with Japan implementing a uniform standard of 0.01 mg/kg for unregulated drugs. In terms of aquaculture water environments, the EU has formulated environmental quality standards (EQSs) for priority antibiotics (e.g., 0.18 μg/L for azithromycin in fresh water), while China currently lacks national unified standards and mostly refers to the predicted no-effect concentration (PNEC) (0.1–3 μg/L for major antibiotic categories) as the ecological safety threshold. For drinking water, the EU mandates that single-antibiotic residues shall not exceed 0.1 μg/L, and total residues shall not exceed 0.5 μg/L. Meanwhile, the WHO recommends a limit of 100 ng/L for norfloxacin and ciprofloxacin. Regarding aquaculture wastewater and soil, the EU aligns with the Water Framework Directive for regulation. Meanwhile, countries such as Canada and Australia adopt standards close to or stricter than international norms. The global regulatory trend is shifting from simple residue control to whole-chain risk management, covering usage, discharge, and residues, with increasing international coordination through frameworks like the Codex Alimentarius Commission [9].

Intensive aquaculture generates high levels of nitrogenous waste (unconsumed feed and animal excretion) [10]. The nitrogen cycle is a microbial mediated process that is fundamental to relieving part of the nitrogen load [11]. The nitrification process can minimize the toxicity of pollutants to aquatic animals. When conditions become anoxic, denitrifying bacteria (DNB) can reduce nitrate to nitrogen gas (N_2_) and accomplish the removal of nitrogen from aquaculture water and prevent potential environmental impacts, such as water eutrophication due to excess nitrogen [12,13]. However, the existence of residual antibiotics may obstruct these essential processes (due to their antimicrobial properties) and prevent microorganisms from coming back, thus changing community structure and metabolic function [14]. Studies have shown that experimental exposure to sulfamethizole (SMZ) modified microbial communities by reducing ammonia-oxidizing bacteria (AOB), and changed denitrifying genus from *Denitromonas* to *Vibrio*, in addition to lowering the abundance of the nitrifying functional genes associated with these genera (*amoA*, *amoB*, and *amoC*) by more than 85% [15,16]. Kalantzi et al. (2021) confirmed that there is a significant negative correlation between DNB copy number and enrofloxacin concentration, demonstrating the ecological consequences of the long-term existence [7].

**Figure 1:**
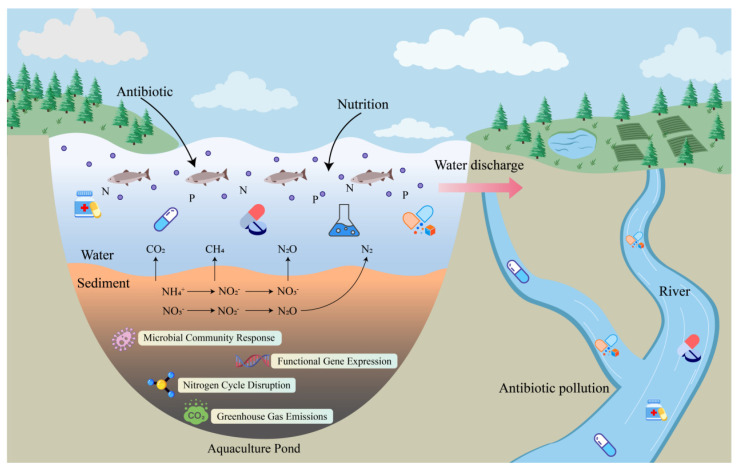
Usage of antibiotics in aquaculture and the impact on nitrogen cycle and GHG emission and surrounding waters.

The nitrogen cycle in aquaculture is intricately associated with greenhouse gas (GHG) emissions [17,18]. Nitrification and denitrification are major sources of nitrous oxide (N_2_O), a potent GHG with nearly 300 times the global-warming potential of carbon dioxide (CO_2_) [19]. AOB produce N_2_O through the oxidation pathway involving hydroxylamine, particularly in conditions of low oxygen or high nitrite [20]. Simultaneously, methanogens, as a group of anaerobes, oxidize organic materials into methane (CH_4_) that would be emitted into the atmosphere [21]. However, the presence of antibiotics may alter the community structure and functional gene abundance of DNB and methanogens, affecting the rate and product distribution of the denitrification process, and may increase the emissions of N_2_O and CH_4_ [22]. It was reported that aquaculture effluent sediments supplemented with oxytetracycline (2 mg/L) under anaerobic incubation, the activity of methanogens was inhibited, and CH_4_ production decreased significantly [23]. The usage of antibiotics in aquaculture and the impact on nitrogen cycle and GHG emission and surrounding waters were shown in Figure 1 Therefore, studying the effects of antibiotics on the nitrogen cycle and greenhouse gas emissions in aquaculture effluents is critical for assessment of ecological risk of antibiotics and for achieving sustainability in aquaculture systems.

Currently, in regard to the research on antibiotic contamination in aquaculture, most of it focuses on the acute toxicity of single antibiotics [24,25,26]. Limited research exists on combined pollution, long-term exposure, or adaptation of microbial communities [27]. This review seeks to systematically summarize the effects of antibiotics on nitrogen cycling and GHG emissions in aquaculture systems. It treats the dual impacts of antibiotics on the microbially mediated nitrogen cycle and GHG emissions as an integrated research focus, clarifying their coupled associations and overcoming the fragmented limitations of existing studies on antibiotics in aquaculture. It delves into the molecular mechanisms through which different types of antibiotics inhibit nitrification/denitrification functional genes (e.g., *amoA* and *amoB*) and reshape microbial communities. Meanwhile, it clarifies the interactive regulatory effects of environmental conditions and co-pollutants. Moreover, this review practically orients the limitations of previous research and outlook on future research frontiers, including combined pollution, long-term exposure, and microbial adaptation. Aligned with the global regulatory trend of whole-chain risk management in aquaculture, it offers direct academic support for the coordinated implementation of antibiotic pollution, nitrogen-removal optimization, and GHG emission reduction in aquaculture systems.

## 2. Antibiotic Pollution in Aquaculture

### 2.1. Antibiotic Use and Residual Concentration in Aquaculture Pond

Antibiotics are frequently detected in aquaculture pond water, discharged effluents, and the surrounding aquatic environment [1]. The commonly detected antibiotics mainly include tetracyclines, sulfonamides, fluoroquinolones, β-lactams, etc. There are common factors among these antibiotics; for example, all are extensively used in aquaculture for the prevention and treatment of fish diseases [28]. They have similar environmental behaviors, such as overuse leading to persistent residues in aquaculture water, sediments, and aquatic products. These residues are difficult to degrade naturally and tend to bioaccumulate [29]. Moreover, nearly all antibiotics possessing antimicrobial properties disrupt the microbially mediated nitrogen cycle and further affect GHG emissions by interfering with related microorganisms [30].

Inland freshwater aquaculture dominates the sector, contributing 62% of global live-weight volume and 75% of global edible weight volume in 2020 [31,32]. Asia is by far the largest aquaculture producer, accounting for 92% of global live-weight production in 2020, and China alone contributes 57% of total aquaculture volume and 59% of global value [32]. Twenty-three types of antibiotics were detected in aquaculture farms on Peninsular Malaysia, with antibiotic resistance genes (ARGs) identified at over 90% of the sampling sites [29]. Twenty-six categories of antibiotics were detected in the surface water of 50 finfish and shellfish aquaculture sites in Bangladesh, among which the peak concentration of sulfadiazine reached 25 μg/L [33]. Su et al. (2025b) reported their presence in both pond water (0.57–1160 ng/L) and sediments (0.41–80.87 ng/g) [34], consistent with the earlier work of Yuan et al. (2019), who identified eight different antibiotics in the water sources, pond water, and sediments of a Chinese shrimp and fish farm [35]. Studies conducted on typical marine aquaculture farms in Southern China revealed the bioaccumulation of 37 different antibiotics, including widespread distribution of residual antibiotics in the marine situation [25]. Antibiotics, including sulfamethoxazole, salinomycin, and trimethoprim, were reported to dissociate into water samples from 0.4 to 36.9 ng/L. Enrofloxacin residues were found and detected between 16.6 and 31.8 ng/g in feed samples, while the antibiotic erythromycin–H_2_O was most frequently detected in sediment samples, at a rate of 0.8 to 4.8 ng/g. Notably, erythromycin–H_2_O was found to be the most abundant antibiotic residue detected in adult Fenneropenaeus penicillatus, ranging from 2498 to 15,090 ng/g [36].

The concentration and distribution of antibiotics in aquaculture systems are affected by many factors, such as the density of farming, dosing frequencies, and the design of the system [37]. There are significant differences in the types and doses of antibiotics used in different regions and aquaculture models [38]. Research by Adenaya et al. directly linked the elevated antibiotic concentrations in coastal waters to aquaculture activities, noting a sharp rise during periods of intensive farming [1]. In intensive aquaculture models, due to high breeding density and high risk of disease occurrence, antibiotics are used more frequently and at relatively higher doses [29,35]. In some extensive aquaculture areas, the use of antibiotics is relatively less, but there are also cases of irregular use [1]. Han et al. investigated the distribution of antibiotics in marine farms around Bohai Bay. They found that the antibiotic concentration level in the culture matrices, including water, sediment/biofilm, and organism, was the highest under greenhouse pond culture mode, and under the industrial recirculating water culture mode, it was the lowest. Antibiotic concentration in culture matrices of fishponds was higher than that of sea cucumber ponds and mollusk ponds [39].

### 2.2. Residues and Risks of Antibiotics in Aquaculture Effluents and Surrounding Environment

Antibiotic residues are common in aquaculture effluents, with their status varying regionally [39,40,41]. Although most detected residues are below maximum residue limits (MRLs), their environmental persistence and potential to induce antibiotic resistance via the food chain threaten aquatic organisms and may impact long-term human health [42]. Evidence from diverse geographical regions underscores the scale of this issue. Antibiotics escape to the ambient environment via effluent discharge, impacting coastal and estuarine environments [34,43]. These pharmaceuticals are now found in many different types of environments, including surface water, drinking water, groundwater, and soils. Some rivers and lakes across China have been shown to contaminate at μg/L and even mg/L levels [44,45]. Kingsbury et al. provided evidence that some antibiotics are persistent near Canadian salmon farms. The most concentrated and abundant antibiotics, as well as organic enrichment, were detected within 200 m of the surface cages; however, residual detectable antibiotics were found up to 1.5 km from the cages [46]. Emamectin benzoate and oxytetracycline residues were detected 4 years and 3 years after the last application of anesthesia, respectively. Li et al. also investigated the distribution, partitioning, and ecological risks of antibiotics in a typical bay of the East China Sea, while Mai et al. examined their occurrence and risks in Hong Lake and its adjacent aquaculture ponds. Both studies were indicative of the potential effects of antibiotic contamination on ecological integrity and human health within aquaculture systems [47,48].

Antibiotics could persist in sediments and water in aquatic environments, especially in intensive aquaculture systems, with organic waste and antibiotics accumulating in sediments [49]. Such accumulation could potentially reduce biodiversity and result in structural changes in benthic ecosystems. The median concentrations of antibiotics that were found in sediments ranged from 0.2 to 54.6 μg/kg, which is much lower than the concentrations of antibiotics observed in soils (0.23 to 157 μg/kg) [50]. Jara et al. demonstrated that the sedimentation of flumequine in Chilean salmon farms was mainly linked to hydrophobicity, while sedimentation of florfenicol was largely linked to surface attachment, not hydrophobicity. As a result, flumequine is likely to be sedimented to a greater degree and have a higher persistence in marine sediments. While it is not known if the residues would persist in the marine environment after the application, the case of flumequine highlights the potential for persistence and warrants further research into the persistence of these compounds in marine systems [51].

In addition to the direct ecological risks posed by prescription antibiotics in the environment, antibiotic residues are also of concern from a public health perspective due to their toxicity to non-target organisms and for promoting antibiotic-resistant bacteria. These residues not only accumulate in sediments, reducing biodiversity and altering benthic ecosystem structures, but also induce bacterial antibiotic resistance and the cross-species transfer of ARGs [52]. They form intractable combined pollution with other pollutants, threatening the integrity of aquatic ecosystems. For human health, these residues bioaccumulate through the food chain, and long-term exposure may lead to potential adverse effects. Furthermore, the dissemination of ARGs poses an irreversible risk to human anti-infective therapy, endangering public health [53]. Moreover, the presence of antibiotics in combination with other pollutants (e.g., organic matter, heavy metals, and microplastics) could pose an acute environmental threat, creating complicated pollution that may not be remediated [23]. Evidence of these potential issues was gathered by Gonzalez-Gaya et al. via their monitoring of flumequine residues in wildlife populations in marine sediments, as well as their observation that florfenicol stimulates an enrichment of ARGs associated with macrolide, tetracycline, and aminoglycoside resistance. Currently, there is still inadequate knowledge regarding the biological processes of antibiotic degradation and antibiotic resistance in aquaculture effluents, thus complicating risk assessment and, potentially, risk management of aquaculture [54].

## 3. Overview of Nitrogen Cycling and Greenhouse Gas Emissions in Aquaculture

The nitrogen cycle, a pivotal process in aquaculture, serves to balance the nitrogen levels in the water and maintain stability within aquaculture production systems [55]. The microbial-driven nitrogen cycle is fundamental for mitigating the toxicity of ammonia nitrogen (NH_3_-N) and preventing eutrophication [13]. However, this vital purification process presents a critical paradox: the same nitrification–denitrification pathways that remove nitrogen are also a primary source of GHG emissions from aquaculture systems [56,57]. This section outlines the key microbial processes involved and establishes their link to GHG production in our discussion of antibiotic-induced disruptions.

### 3.1. Nitrogen-Cycling Processes in Aquaculture Water and Sediments

Aquaculture systems feature large anthropogenic nitrogen input (often as a result of unconsumed feed, or fish excretion, e.g., as NH_4_^+^) [13,55,58,59]. Sediments act as critical bioreactors where redox gradients facilitate successive microbial transformations [9,57,60,61]. The process begins with ammonia oxidation, which is mainly mediated by AOB and ammonia-oxidizing archaea (AOA). Nitrification is aerobic and an essential process in detoxifying ammonia in confined systems, which is vital for maintaining water quality and supporting fish health in intensive systems [62,63].

Nitrate is primarily removed through denitrification, a stepwise anaerobic reduction by heterotrophic denitrifying bacteria (DNB) that converts NO_3_^−^ to N_2_, with N_2_O as an intermediate. Key functional genes include *narG*, *nir*S/*nirK*, and *nosZ* [10]. Additionally, anaerobic ammonium oxidation (anammox) performed by *Planctomycetes* can contribute to nitrogen loss by coupling NH_4_^+^ with NO_2_^−^ to produce N_2_, bypassing the need for organic carbon [11]. Both pathways are crucial for mitigating nitrogen pollution and reducing eutrophication risks in aquaculture effluents. The sediment/overlying-water interface in aquaculture acts as an active hotspot for these shifts in nitrogen speciation, where diffusion of solutes and bioturbation can facilitate the cycling of nitrogen species [22,64]. The microbial consortia mediating these processes—AOA/AOB, NOB, and DNB—are particularly vulnerable to antibiotic exposure, which can disrupt nitrogen removal and promote the accumulation of toxic intermediates such as NH_4_^+^ and NO_2_^−^ [65].

### 3.2. Linkages Between Nitrogen Cycling and Greenhouse Gas Emissions

The Intergovernmental Panel on Climate Change (IPCC) identifies six primary greenhouse gases to calculate the global-warming potential (GWP), among which CO_2_, CH_4_, and N_2_O are the most abundant and widely recognized for their substantial contributions to global warming. Nitrogen transformations in aquaculture are closely tied to the emission of potent GHGs, particularly N_2_O [12,66]. N_2_O, a greenhouse gas that possesses almost 300 times the global-warming potential of CO_2_, is largely produced as a side product of nitrification and an intermediate of denitrification [19]. During nitrification, AOB are able to generate N_2_O when oxidizing hydroxylamine, particularly under low-oxygen or high-nitrite concentrations [20]. However, the denitrification pathway is frequently a larger contributor, wherein intermediate N_2_O is obligatory. The important point that regulates N_2_O emissions is the enzyme N_2_O reductase (*nosZ*), which reduces N_2_O to inert N_2_. Therefore, environmental stressors that can selectively inhibit *nosZ* activity or displace the microbial community away from the effective N_2_O-reducers could result in massive N_2_O accumulation [17,67].

CH_4_ emissions, while not a product of direct nitrogen transformations, are also driven by nitrogen cycling. In aquaculture systems, the metabolic activities of nitrifying and denitrifying microbes alter the redox potential in sediments, thus regulating the niche available for methanogenic archaea [68]. Aerobic conditions at the sediment–water interface support methanotrophic bacteria, which oxidize CH_4_ to CO_2_, thereby reducing emissions [21]. The balance between the microbial community responsible for nitrogen removal and the microbial community that drives GHG production terms is fragile and highly susceptible to external stressors, such as antibiotics [59,69].

The features that make aquaculture ecosystems productive, such as high nutrient loads, organic-rich sediments, and active redox interfaces, also make their nitrogen cycles particularly susceptible to disturbance from antibiotics. This vulnerability is related both to the nature of the systems and, more fundamentally, the microbial ecology within aquaculture ecosystems. This rooted vulnerability serves as the basis for the antibiotic-mediated dysfunctions in nitrogen transformations and greenhouse gas fluxes to be discussed in subsequent sections.

## 4. Impacts and Mechanisms of Antibiotics on Nitrogen Cycling and Greenhouse Gas Emissions

Antibiotic residues emitted through aquaculture wastewater have complex effects on important biogeochemical processes, particularly through the alteration of the structure and function of microbial communities. In this section, we summarize how antibiotics disrupt the nitrogen cycle and affect GHG emissions through microbial community changes, functional gene expression changes, and consequent changes in process efficiencies and gas fluxes.

### 4.1. Alterations in Microbial Community Structure and Functional Gene Expression

Antibiotic exposure results in marked shifts in the composition, diversity, and network complexity of ammonia-oxidizing and -denitrifying microorganisms and their communities. The main outcome would be the large inhibition of nitrifying and denitrifying communities, reducing their metabolic activity and reducing critical functional gene abundance. In addition to being able to select for the fitness of its microbial hosts, the antibiotic-induced selective stress also promotes the emergence of ARGs into microbial communities from its microbial hosts [30]. The interaction between functional gene inhibition and the enrichment of ARGs significantly challenges the capacity of nitrogen removal.

The legacy from aquaculture had a direct influence on microbial activity and strongly affects succession to antibiotic exposures. Metagenomic analyses of sediments treated with oxytetracycline or sulfadiazine showed a significant reduction in sediment microbial diversity in those systems with a 15-year aquaculture history, while younger (5 years) and older systems (more than 30 years) had no statistically significant reductions, thus demonstrating that microbial communities remain tolerant to antibiotic treatments through the aquaculture process [14]. Succession in aquaculture aside, specific antibiotic resistance-gene hosts were identified in sediments from intensive tidal flat aquaculture, including *Candidates Peregrinibacteria* and *Sphingomonadaceae*, while microbial genera of importance to nitrogen cycling, such as *Rhodanobacter* and *Pseudomonas*, were also identified in the water column [49]. Furthermore, the transfer of ARGs is aided by co-stressors associated with hypoxia, promoting greater abundance of antibiotic resistance-gene carriers (e.g., Rhodocyclaceae and *Caldilineaceae*) and horizontal transfer pathways [70].

Critically, the changes to communities are directly linked to the nitrogen cycle. Aquaculture ponds typically have lower abundances of the key denitrifying genes (e.g., *nirK*, *nirS*, and *nosZ*) than natural marshes [18]. Antibiotic exposure exacerbates this issue. Exposure to 2.0 mg/L SMX triggered a sharp decline (85.6–95.3%) in the key ammonia oxidation genes (*amoA*, *amoB*, *amoC*, and *hao*), accompanied by a suppression of Nitrosomonas and a shift in the dominant denitrifying genus from *Denitromonas* to *Vibrio* [15]. Antibiotics also exert an impact on the expression of the *amoA* functional gene in ammonia-oxidizing microorganisms. The effect of high concentrations of fluoroquinolone antibiotics (e.g., enrofloxacin at 10 mg/L) on the expression level of the *amoA* gene results in a significant downregulation and would eventually lead to a decrease in the ammonia oxidation rate, ultimately causing higher levels of accumulated NH_3_-N in aquaculture effluent [71].

Studies have shown that under exposure to low concentrations of tetracycline (0.1–1 mg/L), AOB abundance in the aquaculture simulation system decreased significantly, while AOA abundance increased to a certain extent [72]. This may be attributed to the fact that AOA exhibit stronger tolerance to antibiotics compared with AOB. Antibiotics alter the dominant populations of AOB and AOA [73]. In aquaculture effluents contaminated with sulfonamide antibiotics, the abundance of Nitrosomonas, the original dominant genus of AOB, was decreased, and the relative abundance of some AOB populations with higher tolerance to sulfonamide antibiotics increased [74]. These results emphasize the urgent need to improve our understanding of antibiotic-induced shifts in abundance of N-cycling functional genes and their microbial hosts [75].

### 4.2. Mechanisms of Interference with Nitrogen Transition Processes

The inhibiting impact of antibiotics on the nitrogen cycle is dose-dependent and differs widely amongst antibiotic classes based upon their mechanism of action and the target organisms [76].

The aerobic nitrification process performed by AOB/AOA and NOB organisms appeared to most notably be impacted by antibiotics versus other components of the nitrogen cycle. The mechanism of inhibition differed according to antibiotic class. For example, β-lactams (e.g., penicillin) impair bacterial cell-wall synthesis by binding D-alanine transpeptidase, which blocks the linkages of other D-alanines in the peptidoglycan [77]. This targeted attack ultimately stunts AOB growth and leads to cell lysis, causing a pronounced decline in both growth rate and population density [16]. In contrast, tetracyclines operate through a different mechanism by inhibiting protein synthesis, thus broadly impairing the activity of both AOBs and NOBs [78]. Fluoroquinolones inhibit DNA gyrase, thereby impairing DNA replication. They usually have a greater proportion of effects than other classes of antibiotic; for example, the fluoroquinolone antibiotic norfloxacin (0.5 mg/L) inhibited the AOB activity by as much as 70%. By contrast, erythromycin (a macrolide) at the same concentration would induce a decreased activity of only 30%. A dose of ciprofloxacin at 1 mg/L proved to have a reduction of over 50% in the rate of ammonia oxidation, while 10 mg/L had an effect of nearly 100% [73]. The sulfonamides are competitive antagonists of p-aminobenzoic acid and inhibit folic acid synthesis [79]. AOB, in particular, are sensitive to sulfonamides. NOB possess a greater degree of tolerance to such inhibitors, suggesting a functional group sensitivity to inhibition [15].

The denitrification process was disrupted under anoxic conditions, thus revealing a similar attack from antibiotics. Again, the effect is profound, showing that in treatment with such antibiotics as sulfamethoxazole, chlortetracycline, and ciprofloxacin, there is a profound reduction in nitrate-removal efficiency, with marked nitrite accumulation resulting from such treatment [47,80,81]. This is attributed to failure to activate and express the functional denitrification genes (*narG*, *nirS*, *nirK*, and *nosZ*) and associated with loss of important denitrifying populations such as *Thauera*, *Comamonas*, etc. [80]. The inhibition of the ultimate reaction where the N_2_O is reduced to N_2_ via action of the N_2_O reductase (*nosZ*) is the critical reaction for N_2_O emissions [56].

Different species of nitrifying bacteria may also exhibit varying levels of sensitivity to the same antibiotic. Although both AOB and NOB are involved in the nitrification process, they differ in their tolerance to certain antibiotics. Studies have shown that AOB are highly sensitive to sulfonamide antibiotics—low concentrations of sulfonamide antibiotics can significantly inhibit the growth and ammonia-oxidizing activity of AOB. In contrast, NOB have relatively stronger tolerance to sulfonamide antibiotics; only high concentrations of sulfonamide antibiotics can exert a significant inhibitory effect on their growth and nitrite-oxidizing activity [82]. The anaerobic ammonia oxidation process (carried out by the *Planctomycetes*) is also subject to attack by the antibiotics, but the inhibition by fluoroquinolone antibiotics may be greater in effect than with the tetracycline antibiotics; however, limited systematic research has been conducted to date on this subject matter in aquaculture [83].

### 4.3. Impacts on Greenhouse Gas Emissions

The antibiotic-induced changes in the microbial community and metabolic pathways influence the production and consumption of the potent greenhouse gases N_2_O and CH_4_.

A pivotal environmental consequence of antibiotic disruption is the potential escalation of N_2_O emissions, stemming from altered nitrification and denitrification pathways. During nitrification, AOB produce N_2_O as a by-product of hydroxylamine oxidation, particularly under suboptimal conditions. Antibiotics that inhibit the ammonia mono-oxygenase (AMO) enzyme or hydroxylamine oxidoreductase (HAO) enzyme can stress AOB and may increase the N_2_O yield per unit of ammonia oxidized. Tetracycline at a high concentration (5 mg/L) was shown to inhibit AOB activity but stimulate N_2_O production, probably because of its interference with normal metabolic pathways [17]. The shift in the AOB community structure to include strains with higher N_2_O-yielding potential (e.g., Nitrosomonas europaea) under antibiotic stress can compound the emissions [20]. This suggests that even when overall nitrification rates are suppressed, the process may become a more potent source of this potent greenhouse gas.

Denitrification is the main pathway for the production of N_2_O. Antibiotics such as sulfamethoxazole inhibit the N_2_O reductase (*nosZ*) enzyme activity and create a bottleneck that results in N_2_O accumulation instead of its reduction to inert N_2_ [15]. Environmental conditions modify this effect; for example, low dissolved oxygen can operate in synergy with antibiotics to further enhance N_2_O emissions [19].

Antibiotics also affect methanogenesis and methanotrophy, thus accelerating or inhibiting CH_4_ emissions. Methanogenic archaea are susceptible to antibiotic inhibition, which inhibits CH_4_ production. For instance, in anaerobic incubations of aquaculture sediments, oxytetracycline (2 mg/L) suppressed methanogenic activity associated with mollusks and reduced CH_4_ output, possibly due to the inhibition of vital enzymes [21]. Tetracycline demonstrates more potent inhibition on methanogenesis than sulfonamides—evidenced by IC_50_ values as low as 37.0 mg/L—primarily through suppressing acetogenesis and subsequently altering methanogenic community structure and dominant pathways over prolonged exposure [74]. On the other hand, methanotrophic bacteria oxidized CH_4_ to CO_2_ and act as a biological sink for methane. However, antibiotic exposure may also blunt methanotrophic activity, thereby reducing the oxidative consumption of CH_4_. Thus, the suppression of methanotrophic microbes may lead to an increase in net methane emissions, specifically at the sediment–water interface [84].

### 4.4. Conceptual Synthesis of Interacting Mechanisms

**Figure 2:**
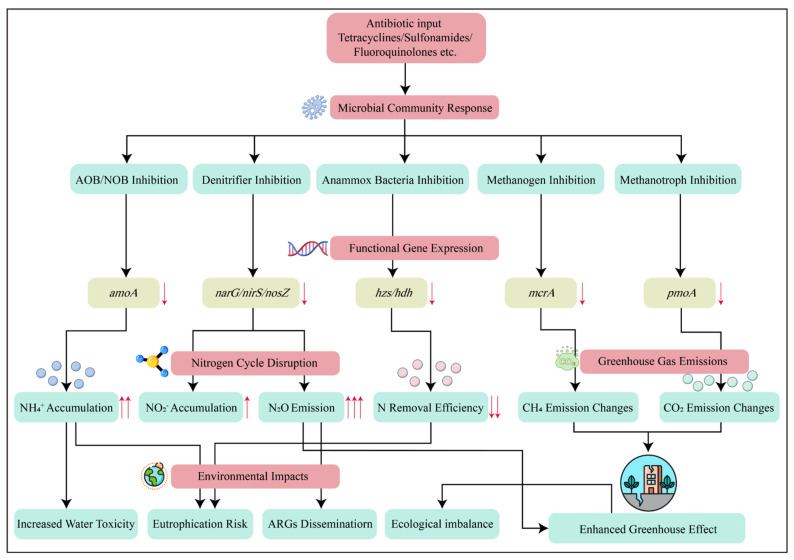
Impacts and mechanisms of antibiotics on nitrogen cycling and greenhouse gas emissions.

The overall consequences of antibiotics on nitrogen cycling and GHG emissions can be viewed as a cascade of perturbations occurring at multiple different levels which were demonstrated in Figure 2. Firstly, antibiotics directly inhibit important functional microorganisms, such as AOB, NOB, denitrifiers, anammox, and methanogens. Secondly, antibiotics inhibit the expression of some important functional genes, such as *amoA*, *nxrB*, *nirS*, *nosZ*, and *mcrA*. Moreover, the nitrogen cycle is decoupled in the presence of antibiotics, leading to reduced nitrogen-removal efficiency; buildup of toxic nitrogen intermediates, such as NH_4_^+^ and NO_2_^−^; and altered ratios of gaseous end-products. Lastly, antibiotics cause the dysregulation of nitrification/denitrification, especially *nosZ* inhibition, leading to increased N_2_O emissions, and the imbalance between methanogenesis and methanotrophy alters net CH_4_ flux. This conceptual model underscores that the ultimate magnitude of GHG emissions is not determined by one single mechanism but by the interplay of all of these disrupted layers.

## 5. The Synergistic Effects of Antibiotics and Co-Contaminants on the Nitrogen Cycle

### 5.1. Interaction Between Carbon-to-Nitrogen Ratio (C/N) and Antibiotics in Aquaculture

The C/N ratio serves as an important ecological factor that can fundamentally modulate the ecological effects of antibiotics used in aquaculture systems. Rather than having an isolated influence, C/N conditions alter microbial community structure and metabolism, resulting in an eventual alteration in the level of disturbance, either severe or minimal, for nitrogen cycling, thereby determining the nitrogen-removal efficiency and stability of the system [85].

The effect the C/N ratio has on nitrification during antibiotic exposures stems from the changes in microbial competition [81]. In conditions of low C/N ratio (typically C/N < 5), nitrification appears to exhibit improved resilience to antibiotic inhibition. This occurs because limited organic carbon suppresses heterotrophic microorganisms’ growth, reducing their competitive advantage and enabling autotrophic nitrifying bacteria (AOB/NOB) to dominate [86]. Supporting this, Li et al. observed that in a mariculture system containing SMX (500 μg/L), when the C/N ratio was 3, the abundance of AOB was 42% higher and the activity of AMO was 38% higher than when the C/N ratio was 10. The low C/N ratio improved the tolerance of nitrifying bacteria to SMX by reducing competition from heterotrophic bacteria, maintaining the NH_4_-N-removal rate at above 85% [15]. In addition, it is reported that in the presence of tetracycline (200 μg/L), the abundance of NOB in the group with a C/N ratio of 2 was 57% higher than that in the group with a C/N ratio of 8, and the inhibition rate of NOR activity was reduced by 29% [87]. Additionally, the activity of antioxidant indicator of autotrophic nitrifying bacteria under low C/N conditions was 1.6 times that of the high C/N group, thus effectively mitigating the oxidative stress induced by tetracycline [88].

In contrast, high C/N ratios aggravate antibiotic inhibition of the nitrification process. These elevated carbon conditions stimulate heterotrophic growth, which exacerbates the competition for dissolved oxygen and biofilm space, with nitrifiers ultimately reducing the abundance of AOB/NOB [89]. Furthermore, certain metabolites generated by heterotrophic bacteria may amplify the biological toxicity of antibiotics. This further represses AMO and NOR activity, ultimately constraining the conversion of NH_3_-N [90].

The moderating role of the C/N ratio in denitrifying under antibiotic stress can be conceptualized as a battle for resources, with carbon availability dictating the defensive capacity of DNB. Under high C/N ratios, a substantial organic carbon load is not only required as the electron donor for the reduction of NO_3_^−^ and NO_2_^−^ but also as a metabolic construct that enables effective resistance. With sufficient organic carbon, bacteria can redirect energy toward the synthesis of protective extracellular polysaccharides (EPSs) and possibly even antibiotic degrading-enzyme productions [91], thereby alleviating the inhibitory effect of antibiotics [92]. This increased resiliency, practically, is an important consideration when measured by Zhang et al., who found that their system had a denitrification rate that was 2.8 times higher, and nitrate reductase (NR) and nitrite reductase (NIR) enzyme activities that were 65% and 58% higher, respectively, with a C/N of 10 than at a C/N of 2 in their experimental system with enrofloxacin (10 mg/L) [93].

Alternatively, low C/N ratios (<3) worsen antibiotic inhibition of denitrification. The limitation in organic carbon itself reduces the synthesis of NR and NIR enzymes in DNB, and the insufficient carbon source means that electron donors are diminished. The addition of quinolone and sulfonamide antibiotics would add to the inhibition of denitrification through inhibition of the respiratory chain of DNB and damage to intracellular enzyme structures, thereby preventing DNB from producing sufficient NR, NIR, and EPS [89]. This creates near-collapse of the microbial community and its functionality, with evidence of this shown by a 53% lower abundance of denitrifiers such as Pseudomonas, and a 4.1-fold greater accumulation of NO_3_^−^ exhibited with a C/N of 3 with sulfadiazine compared to the C/N of 8 [93]. Importantly, the lack of carbon substrates also impairs the synthesis of antioxidants such as glutathione. The production of ROS further inhibits the cellular structures necessary for enzymatic activity in DNB, resulting in cyclical functional inhibition of denitrification [94].

### 5.2. Interaction Between Antibiotics and Co-Pollutants

Antibiotics in real aquaculture settings seldom exist independently; rather, they coexist and interact with other pollutants, such as heavy metals, engineered nanoparticles (ENPs), and microplastics. Such interactions can greatly modify the antibiotics’ environmental behavior, bioavailability, and ecotoxicity [95,96].

Heavy metals are common co-pollutants in aquaculture systems that are derived from feed formulations and antifouling agents [97]. Interaction between antibiotics and heavy metals can be synergistic or antagonistic [98]. Heavy metals would cause cell membrane damage, increasing the permeability of antibiotics into the microbial itself, thereby augmenting inhibitory action [99]. Additionally, antibiotics would bind heavy metal ion(s) via functional groups (e.g., hydroxyl and carbonyl), thereby affecting the overall toxicity of the mixture and the bioavailability of both the integer pollutants and the antibiotics. Antibiotic–metal complexes may also electrostatically interact with charged microbial cell membranes, with the possibility of further enhancing disrupting effects [43,100]. Li et al. investigated that the combination of tetracycline and Cu^2+^ exhibits a stronger inhibitory effect on nitrifying bacteria than either pollutant alone [37]. Conversely, antibiotics may compete with heavy metals for adsorption sites on organic matter or minerals, increasing the bioavailability of both pollutants [64]. The coexistence of sulfadiazine and lead (Pb) inhibits the abundance of nitrification functional genes and nitrifying bacteria in surface sediments, resulting in increased concentrations of NH_4_^+^ and NO_2_^−^ in the overlying water [101].

Microplastics are ubiquitous in aquaculture environments due to the use of plastic fishing gear and feed packaging [102,103]. Their interaction with antibiotics is mainly reflected in adsorption and transport process. Microplastics, especially those with hydrophobic surfaces, can adsorb hydrophobic antibiotics and transport them over long distances, expanding the scope of antibiotic pollution [41,104]. On the other hand, antibiotics adsorbed on microplastics are released slowly into the water, resulting in chronic, low-level exposure of microorganisms to antibiotics, which is more likely to induce the development of microbial resistance compared to short-term high-concentration exposure [105]. As a result, microplastics can extend the persistence of antibiotic toxicity in the ecosystem [96].

The advent of ENPs in water treatment and disease control is rising in aquaculture, causing their coexistence with antibiotics [106]. ENPs have high specific surface area and numerous sites for adsorption, allowing for antibiotic sorption through electrostatic forces or hydrophobic interactions [107]. This adsorption reduces the free antibiotic concentration in water, thus relieving their inhibitory impact on microorganisms. However, desorption of the adsorbed antibiotics can occur under certain conditions that significantly raise the effective concentration of the antibiotics, e.g., a change in pH or ionic strength [108]. Moreover, ENPs and antibiotics have interactive toxicity. ENPs stimulate oxidative-stress induction that proffers specified biological toxicity. When paired with antibiotics, the ENPs could possibly increase the toxicity of antibiotics when this is applied to microorganisms. TiO_2_ nanoparticles improved the infiltration of quinolone antibiotics into bacteria, leading to enhanced inhibition of DNB [109].

## 6. Limitations of Current Research, and Future Prospects

**Figure 3:**
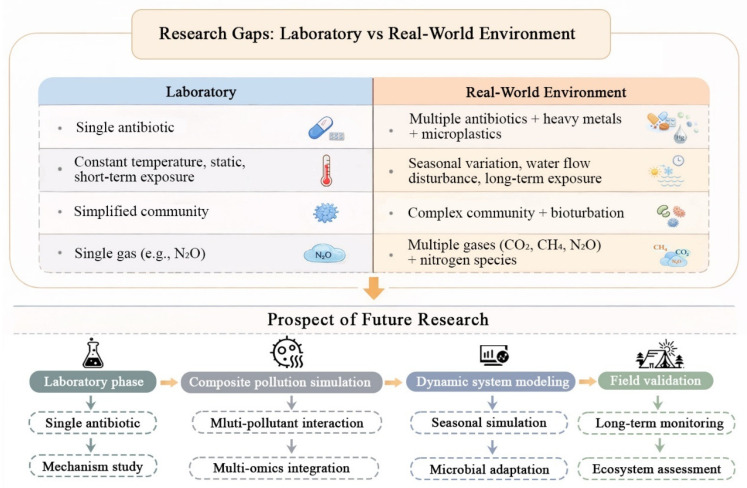
Limitations of current research, and future prospects.

Figure 3 indicated that although recent studies indicate that antibiotics change nitrogen cycling and greenhouse gas fluxes in aquaculture systems, they also come with caveats related to the simplified experimental designs in much of the existing research, as well as limited mechanistic understanding and ambient complexity [110].

### 6.1. Disconnect Between Laboratory Models and Reality

Most current studies consider single antibiotics, or simple antibiotic and single heavy metal combinations, which are quite different from the polluted mixtures that characterize real aquaculture systems [104]. In actual aquaculture waterbodies, antibiotics and co-contaminants may alter nitrogen-cycle functions via toxicity synergy or toxicity antagonism. Existing research lacks adequate quantitative assessment and mechanistic dissection of these intricate interactive effects [76]. Furthermore, most studies monitor N_2_O or CH_4_ emissions in isolation, overlooking both the contribution of CO_2_ emissions and the indirect regulatory role of carbon–nitrogen-cycle coupling in greenhouse gas dynamics [27]. The inability to evaluate the impacts of antibiotics more holistically on the climatic footprint of aquaculture systems is currently a major gap. Furthermore, antibiotics could indirectly influence nitrogen cycling by changing microbial carbon metabolism, yet existing studies do not incorporate carbon and nitrogen cycling [24].

Most studies utilize short-term static laboratory simulations, which ignore long-term dynamic environmental factors that exist as part of the real-world aquaculture, including seasonal temperature fluctuations, stratification of dissolved oxygen, and community interactions of biotic populations. Within natural ponds, the water temperature varies seasonally, and as the temperature increases, microbial metabolic activity is largely temperature-dependent [42]. As such, static constant-temperature trials cannot reflect the real-world seasonal changes inherent to real-world systems [111]. Furthermore, laboratories typically employ static sediment–water-stratified designs, but, in reality, aquaculture ponds experience disturbance (e.g., aeration and water exchange), which subsequently alters the rate of migration of antibiotics at the sediment–water interface, in addition to the exposure concentration for microorganisms—ultimately diminishing the ecological significance of experimental outcomes [112].

To address the limitations, efforts should focus more on complex pollution scenarios and quantify multi-pollutant interactive effects. Firstly, researchers need to design multi-pollutant-exposure experiments encompassing multiple antibiotic classes and multiple heavy metals, plus aquaculture-derived pollutants. Secondly, researchers should employ response surface models to quantify the synergistic/antagonistic coefficients of different pollutants and identify critical concentration thresholds at which complex pollution impacts nitrogen-cycle functions [113]. Meanwhile, researchers need to establish dynamic in situ experimental systems to enhance ecological relevance and conduct long-term partial in situ or in situ experiments, for example, constructing simulated aquaculture ponds and monitoring them over a full culture cycle. Lastly, researchers should integrate dynamic factors, including temperature, dissolved oxygen, and biotic communities (benthic fauna and submerged plants), to analyze the migration and transformation of antibiotics at the water–sediment–organism interface, as well as their long-term cumulative effects on the nitrogen cycle [114,115].

### 6.2. Incomplete Understanding of Underlying Mechanisms

Observational data have linked antibiotic use to observations such as reduced nitrogen-transformation efficiency and elevated CH_4_/N_2_O emissions, molecular-level insights into the metabolic pathways of key functional microbial groups remain lacking [27]. For example, the molecular signaling pathways through which antibiotics inhibit AMO activity in AOA and AOB, thereby blocking nitrification, remain uncharacterized [116]. In addition, most existing studies rely on functional gene microarrays or quantitative PCR to measure changes in specific gene abundance, but they fail to integrate proteomic and metabolomic data to capture dynamic shifts in metabolic pathways under antibiotic exposure [27,85].

The potential role of horizontal gene transfer in antibiotic-driven greenhouse gas emissions is also insufficiently understood. Although antibiotics can promote the spread of ARGs via mobile genetic elements (e.g., upregulating integron *intI1* genes), their impact on the transfer of nitrogen cycle functional genes is virtually unexplored [53,116]. Finally, research on the cross-scale mechanism by which the combined action of antibiotics and other environmental stressors, like heavy metals and organic matter, in aquaculture waterbodies modulates the long-term adaptability of functional microbial communities via epigenetic modifications (for example, DNA methylation and histone acetylation) is still in its infancy [27,104,117].

More research should focus on molecular mechanisms and move beyond correlational studies to gain a mechanistic understanding of how antibiotics inhibit microbial nitrogen metabolism. Thus, there is a need for integrated meta-omics (metagenomics, metatranscriptomics, and metabolomics) to define the exact inhibition of specific enzymes, such as ammonia AMO and N_2_O reductase (*nosZ*), and if/how they change regulatory pathways in ways that lead to the formation of toxic intermediates and greenhouse gases such as N_2_O [27,73].

## 7. Implementing Sustainable Practices and Regulations for Antibiotic Management in Aquaculture

Scientific research on the effects of antibiotics on the nitrogen cycle and greenhouse gases must be implemented through management practices that protect against ecological risk and promote sustainable aquaculture. This section outlines practical strategies to reduce antibiotic reliance, discusses regulatory and policy measures, and proposes an integrated framework for chemical risk control.

### 7.1. Sustainable Aquaculture Management Practices

The adoption of improved aquaculture practices can significantly decrease antibiotic dependence and minimize their environmental release. Key strategies focus on enhanced husbandry, health management, and system design. Firstly, optimizing husbandry conditions, like maintaining good water quality, implementing appropriate stocking densities, and providing balanced nutrition, would strengthen host immunity and reduce disease incidence, thereby lowering the need for antibiotic use [28]. Complementing this, robust biosecurity measures, including controlled water sourcing, systematic disinfection of equipment, and managed effluent discharge, are essential to prevent pathogen introduction and limit the spread of antibiotics into surrounding ecosystems [8]. Also, system-level innovations offer promising pathways. Integrated Multi-Trophic Aquaculture (IMTA) systems, which co-culture species from different trophic levels (e.g., finfish, shellfish, and seaweed), enhance nutrient recycling, reduce waste accumulation, and thus, increase ecosystem stability. By diversifying microbial communities and lowering nitrogen loads, such systems may also buffer against antibiotic-induced disruptions [118].

### 7.2. Regulatory and Policy Approaches for Antibiotic Stewardship

Effective governance is fundamental to curbing antibiotic overuse and environmental contamination. Regulatory frameworks must be comprehensive, enforceable, and adaptive. Many countries have developed policies and regulations governing antibiotic usage in aquaculture; generally, these include banned antibiotics and/or the maximum residual limits (MRLs) of specific substances in aquaculture products, as well as systems and structures to support regulatory enforcement [119]. One the other hand, monitoring and reporting systems need to be formed. Developing national and regional surveillance programs to track antibiotic usage, residue levels in products and environments, and antimicrobial resistance (AMR) trends can inform policy adjustments and risk assessments [120]. Meanwhile, as aquaculture is a global industry, international collaboration is needed to harmonize regulations, share best practices, and align with guidelines from organizations [121].

### 7.3. Integrated Sustainability Framework Incorporating Chemical Risk Control

A comprehensive view of sustainable aquaculture necessitates the incorporation of chemical risk assessment and management throughout the design and operation of aquaculture systems. By applying environmental risk assessment (ERA) models, the environmental impact of antibiotics on nitrogen-cycling microbes (nitrifying bacteria) and greenhouse gas (GHG) emissions can be evaluated. The ERA models must take into consideration the persistence, availability of bioactive compounds (bioavailability), and interaction of antibiotics with other chemical pollutants in order to increase the precision of risk evaluation and improve decision-making based on available evidence [122]. Furthermore, encouraging the development and use of environmentally friendly alternatives such as phage therapy, probiotics, immunostimulants, and herbal extracts will further reduce the ecological impact of managing disease [123]. By employing a life-cycle assessment (LCA) approach, aquaculture producers will be able to quantify their environmental impacts due to antibiotic use through all stages of production, including feed inputs through effluents discharged from operations. A systems analysis of the LCA will allow for the identification of target areas for intervention to reduce overall environmental impact [124,125].

In summary, transitioning toward sustainable aquaculture necessitates a multi-faceted strategy that couples scientific insight with practical management, rigorous regulation, and stakeholder engagement. Integrating improved practices, stringent antibiotic stewardship, and proactive chemical risk control into a cohesive framework will enable the sector to minimize its environmental footprint while ensuring long-term ecological and economic viability [126].

## 8. Conclusions

This review synthesizes evidence that antibiotic residues in aquaculture systems significantly disrupt microbially driven nitrogen cycling and exacerbate greenhouse gas emissions. By altering microbial community structure, inhibiting functional gene expression (e.g., *amoA* and *nosZ*), and interfering with nitrification and denitrification processes, antibiotics reduce nitrogen-removal efficiency, promote the accumulation of toxic nitrogen intermediates, and enhance the release of N_2_O and CH_4_. These impacts are further modulated by environmental factors such as the C/N ratio and synergistic interactions with co-occurring pollutants, like heavy metals and microplastics. Current research remains constrained by simplified experimental designs focused on single contaminants, highlighting a critical gap in understanding complex pollution scenarios and underlying molecular mechanisms. Moving forward, a dual focus is imperative: advancing fundamental knowledge through integrated multi-omics and in situ monitoring to elucidate mechanistic pathways, and translating this knowledge into integrated management strategies. Effective solutions will require the concurrent development of antibiotic mitigation practices, enhanced nitrogen-removal technologies, and targeted greenhouse gas-reduction measures to ensure the ecological sustainability and long-term viability of aquaculture ecosystems.

## Data Availability

No new data were created or analyzed in this study. Data sharing is not applicable to this article.
